# MicroRNAs as Orchestrators of Immune Responses to Bacterial Infection

**DOI:** 10.3390/microorganisms14030515

**Published:** 2026-02-24

**Authors:** Lingjie Li, Yitao Xiang, Yujie Cai, Fangzhen Luo

**Affiliations:** School of Pharmaceutical Science, Hengyang Medical College, University of South China, Hengyang 421001, China; 15211544672@139.com (L.L.); 15399911387@163.com (Y.X.); 13085119661@163.com (Y.C.)

**Keywords:** miRNA, host–pathogen interactions, bacterial pathogens, immune regulation, infection mechanisms

## Abstract

MicroRNAs (miRNAs) are essential post-transcriptional regulators of gene expression and have emerged as key modulators of host–pathogen interactions during bacterial infection. In this narrative review, we synthesize recent experimental and mechanistic evidence on how infection-responsive miRNAs shape innate and adaptive immunity, focusing on four representative pathogens: *Salmonella*, *Listeria monocytogenes*, *Mycobacterium tuberculosis*, and *Helicobacter pylori*. We highlight major miRNA-regulated signaling modules, including TLR/NF-κB, JAK–STAT, autophagy, immunometabolic reprogramming, and extracellular vesicle mediated intercellular communication, and summarize experimentally validated miRNA–target interactions that calibrate immune activation thresholds and inflammatory outcomes. Accumulating evidence indicates that miRNAs not only fine-tune host defense programs by controlling immune-related gene expression and immune cell activation, but can also be exploited by bacterial pathogens to suppress antimicrobial signaling and promote intracellular survival or persistent colonization. Collectively, these findings position miRNAs as a critical regulatory layer linking immune signaling networks to infection outcomes and underscore their translational potential as biomarkers and host directed therapeutic targets, while remaining grounded in current experimental evidence.

## 1. Introduction

In recent years, microRNAs (miRNAs), a class of non-coding small RNAs approximately 20–22 nucleotides in length, have been recognized as key regulators in the post-transcriptional gene expression network. They exert their effects by targeting the 3′ untranslated region (UTR) of mRNA, leading to translational repression or degradation. MiRNAs play a wide range of roles in regulating inflammatory responses, immune cell differentiation, metabolic reprogramming, and more. These functions make miRNAs crucial players in the host’s immune response to bacterial infections [1]. Extensive studies have shown that the expression profiles of miRNAs in host cells are significantly reshaped following infection with common bacteria, including Gram-positive bacteria such as *Staphylococcus aureus* and *Listeria monocytogenes*, as well as Gram-negative or intracellular pathogens such as *Salmonella* enterica, *Pseudomonas aeruginosa*, *Mycobacterium tuberculosis*, and *Helicobacter pylori*. For instance, miR-155, miR-146, miR-21, miR-223, miR-5112, and miR-26a have been found to be upregulated or downregulated in various infection models. They influence pathogen clearance or inflammatory dysregulation by regulating classical inflammatory pathways such as TLR/NF-κB, phagocytosis-related genes, and cellular metabolic pathways [2,3].

MiRNAs regulate both innate and adaptive immunity: On one hand, they adjust cytokine release and activation of macrophages and dendritic cells by directly targeting inflammatory mediators or upstream signaling molecules, thereby affecting bacterial clearance efficiency and the extent of tissue inflammation [4,5]. On the other hand, miRNAs alter macrophage phagocytic and bactericidal abilities by modulating host metabolism (e.g., SIRT6/HIF-1α, PPARα/ABCA1), providing or depriving intracellular pathogens (such as *M. tuberculosis*, *L. monocytogenes*) with a survival niche [6,7]. Moreover, recent studies suggest that miRNAs carried by exosomes or extracellular vesicles can be transmitted between cells, participating in bidirectional signaling exchanges between the host and microbiota, emerging as a novel regulatory mechanism and potential source of biomarkers for local/systemic immune microenvironment modulation [8].

It is important to note that pathogens are not passive recipients of host regulation. Increasing evidence suggests that certain bacteria can actively manipulate host miRNA expression (or influence miRNA processing and sorting through secreted factors or effector proteins), suppressing host-beneficial antimicrobial miRNAs or upregulating miRNAs that favor their own survival. This enables immune evasion and the establishment of chronic infections [9]. These dynamic bidirectional interactions make miRNAs both key executors of immune regulation and important determinants of infection outcomes (clearance, persistent infection, or inflammatory damage) [10,11].

Based on the above background, this review focuses on the molecular mechanisms of miRNA-mediated immune responses in common bacterial infections, with an emphasis on how miRNAs regulate downstream inflammatory signals (e.g., TLR/NF-κB, IKK complexes) [12]. By integrating recent studies and reviews, we aim to provide a theoretical and experimental foundation for understanding the role of miRNAs in infection immunology and their potential as clinical biomarkers or therapeutic targets [13].

Importantly, accumulating evidence suggests that miRNAs do not act as simple on-off switches of inflammation during bacterial infection. Instead, they function as regulatory rheostats that calibrate immune activation thresholds across different pathogens, cell types, and infection stages. Within this framework, a limited set of core miRNAs repeatedly targets central immune signaling hubs, whereas additional context-dependent miRNAs fine-tune local responses in a pathogen- and tissue-specific manner. The objective of this review is to integrate experimentally validated miRNA–target–pathway mechanisms that shape host immune responses across representative bacterial pathogens, including *Salmonella*, *Listeria monocytogenes*, *Mycobacterium tuberculosis*, and *Helicobacter pylori*. Unlike previous reviews that mainly catalog miRNA expression changes or focus on single pathogens, we propose a unified pathway-oriented framework in which miRNAs function as hierarchical immune rheostats. This framework highlights conserved immune hubs and pathogen-specific adaptations with translational implications for biomarker discovery and host-directed therapy.

To ensure that this review is grounded in robust and biologically meaningful evidence, literature was retrieved primarily from PubMed, Web of Science, and Google Scholar, covering studies published between January 2000 and January 2025, with emphasis on advances from the last 5–10 years. The search was not intended to be exhaustive but aimed to capture representative mechanistic and translational studies. Search terms combined “microRNA” or “miRNA” with keywords related to bacterial infection and immune regulation (e.g., “host–pathogen interaction”, “TLR/NF-κB”, “JAK–STAT”, “autophagy”, “metabolic reprogramming”, and “exosome”), together with pathogen-specific terms (e.g., *Salmonella*, *Listeria monocytogenes*, *Mycobacterium tuberculosis*, and *Helicobacter pylori*). Studies were prioritized if they provided experimentally validated miRNA–target interactions (e.g., reporter assays, gain- or loss-of-function analyses), clear immune-related functional evidence, and defined downstream signaling pathways. In vivo validation and patient-derived samples were emphasized. In contrast, purely predictive computational analyses without functional confirmation were cited only as supportive evidence, and studies relying on heavily engineered bacterial mutants were excluded when such manipulations substantially altered physiological host–pathogen interactions.

## 2. MiRNA Biogenesis and Functions

**Figure 1:**
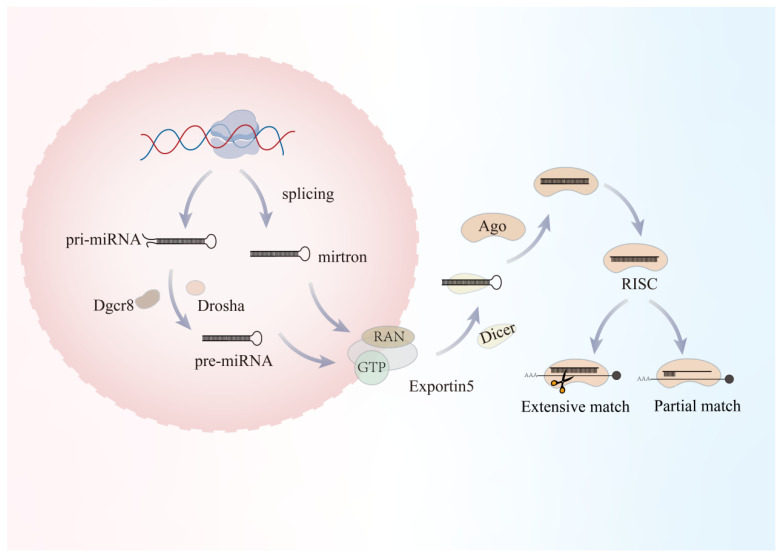
Biogenesis and post-transcriptional regulatory mechanisms of microRNAs. MicroRNA biogenesis begins in the nucleus with the transcription of primary microRNA (pri-miRNA), which is processed by the Drosha-DGCR8 complex to generate precursor microRNA (pre-miRNA). In parallel, certain intronic miRNAs can be produced through the mirtron pathway following splicing. Pre-miRNA is exported to the cytoplasm via the Exportin-5/Ran-GTP complex and further cleaved by Dicer to produce a mature miRNA duplex. The guide strand is incorporated into the Argonaute-containing RNA-induced silencing complex (RISC), where it mediates gene silencing through extensive or partial base pairing with target mRNAs, leading to mRNA degradation or translational repression.

MiRNA genes are transcribed mainly by RNA polymerase II (Pol II), producing primary transcripts (pri-miRNAs) that can be thousands of nucleotides long [14,15]. Pri-miRNAs share canonical mRNA features, including a 5′7-methylguanosine cap (m7G cap) and a 3′ polyadenylated tail (poly(A) tail) [14,15,16]. Figure 1 was created with BioGDP.com to illustrate the biogenesis and post-transcriptional regulatory mechanisms of miRNAs.

In the nucleus, pri-miRNAs undergo the first cleavage step catalyzed by the microprocessor complex [14,17,18], which consists of Drosha (a class II RNase III endonuclease that forms the catalytic core) and DGCR8 (a double-stranded RNA-binding protein) [14,18,19]. This complex recognizes and cleaves pri-miRNAs to generate a 60–70 nt hairpin precursor termed the precursor miRNA (pre-miRNA) [14,15,17]. Newly produced pre-miRNAs must then be exported to the cytoplasm to complete maturation. Assisted by high nuclear RanGTP, the nuclear export receptor Exportin-5 (XPO5) specifically binds the 3′ overhang and the double-stranded stem of the pre-miRNA, forming a pre-miRNA/XPO5/RanGTP ternary complex [17,20,21]. This complex translocates through the nuclear pore complex (NPC) into the cytoplasm, where RanGAP (a GTPase-activating protein) promotes hydrolysis of RanGTP to RanGDP, triggering a conformational change in XPO5 and releasing the pre-miRNA.

In the cytoplasm, pre-miRNAs are captured by another RNase III enzyme, Dicer [14,17,22]. Through its PAZ domain, Dicer recognizes the 3′overhang and the 5′phosphate of the pre-miRNA and removes the terminal loop [23]. Dicer typically functions in complex with the double-stranded RNA-binding proteins TRBP (TAR RNA-binding protein) or PACT (protein activator of PKR) [21,24,25]. TRBP not only facilitates substrate recruitment but also modulates Dicer cleavage precision, thereby influencing length heterogeneity of mature miRNAs (isomiRs) [24,25,26]. The cleavage product is a 22 bp RNA duplex comprising a guide strand and a passenger strand [16,20,24]. The duplex is subsequently loaded onto an Argonaute (Ago) protein to form the RNA-induced silencing complex (RISC) [15]. Importantly, not both strands are retained: the strand whose 5′ end is less stably paired (i.e., weaker hydrogen bonding, typically A/U-rich) is preferentially selected as the guide strand (the mature miRNA), whereas the passenger strand is displaced and degraded [15,16,20].

Beyond the canonical pathway, multiple alternative routes have evolved to bypass Drosha or Dicer; these non-canonical miRNAs often play critical roles in specific tissues or developmental stages [14,27,28]. For example, mirtrons are intron-derived miRNAs whose biogenesis completely bypasses the Drosha/DGCR8 complex [21,29,30,31]. Mirtron precursors are generated directly through mRNA splicing: after short introns are excised by the spliceosome [32], the resulting lariat is linearized by the debranching enzyme DBR1 and folds into a pre-miRNA-like hairpin, which then enters the canonical nuclear export and Dicer processing steps [26].

Once RISC is loaded with a mature miRNA, it searches for target mRNAs via complementary base pairing [33]. The regulatory outcome largely depends on the degree of complementarity between the miRNA and its target. Extensive matching can activate the endonucleolytic “slicer” activity of Ago2, leading to direct cleavage of the mRNA near the center of the paired region. In contrast, partial matching-typically involving imperfect pairing between the miRNA seed sequence and sites within the 3′ UTR-recruits cofactors that promote mRNA deadenylation, decapping, and subsequent decay or alternatively suppress translation directly [14,16,24].

## 3. Regulation of Immune Responses by MiRNAs

In the following sections, we summarize experimentally validated miRNA–target interactions that regulate host immune responses during bacterial infection, with emphasis on four representative pathogens: *Salmonella*, *Listeria monocytogenes*, *Mycobacterium tuberculosis*, and *Helicobacter pylori*. We focus on miRNA-regulated signaling modules that recurrently converge on key immune hubs, including TLR/NF-κB signaling, cytokine-associated pathways, autophagy, and immunometabolic circuits [34]. Where available, we also highlight evidence supporting extracellular vesicle mediated miRNA communication and pathogen-driven manipulation of host miRNA networks [35].

Subsequent studies have shown that miRNA-mediated immunoregulation is a common response following pathogen infection [36]. In the following sections, we summarize major advances in this field according to pathogen type, with an emphasis on bacterial species. 

### 3.1. MicroRNA-Mediated Fine-Tuning of TLR/NF-κB Signaling and Exosomal Immune Communication During Salmonella Infection

*Salmonella* is a Gram-negative facultative intracellular pathogen that invades host cells through type III secretion systems encoded on pathogenicity islands. Following internalization, *Salmonella* delivers a repertoire of effector proteins that interfere with phagosome maturation and lysosomal function, thereby promoting intracellular survival and replication within macrophages. Clinically, infection commonly manifests as self-limiting gastroenteritis characterized by diarrhea, abdominal pain, fever, and vomiting, but, in susceptible individuals, may progress to invasive diseases including bacteremia, sepsis, and meningitis. During infection, *Salmonella* robustly activates host innate immune signaling pathways, including nuclear factor κB (NF-κB), mitogen-activated protein kinase (MAPK), Janus kinase–signal transducer and activator of transcription (JAK–STAT), and interferon-related cascades. Increasing evidence indicates that these responses are tightly regulated by host microRNAs (miRNAs), including miR-143 and members of the let-7 family [37,38,39,40]. Rather than functioning as direct antibacterial effectors, most *Salmonella*-responsive miRNAs act as modulators that calibrate the magnitude and duration of innate immune signaling, particularly within Toll-like receptor (TLR)–NF-κB centered pathways. Evidence for *Salmonella*-associated miRNA regulation is largely derived from in vitro macrophage/epithelial infection systems and validated in multiple in vivo models (mouse, pig, and chicken), providing a comparative framework for mechanistic interpretation across host species.

During *Salmonella* infection, miRNAs play a critical role in regulating TLR–NF-κB–centered inflammatory signaling. Multiple studies have demonstrated that miRNAs induced or suppressed during infection fine-tune inflammatory output by modulating this pathway. Schulte and colleagues reported that *Salmonella* infection or LPS stimulation consistently downregulated members of the let-7 family in murine macrophages, human epithelial cells, and murine infection models [41]. This regulation was largely dependent on TLR4-mediated LPS signaling. Functionally, let-7 directly binds the 3′ untranslated regions (UTRs) of IL6 and IL10 mRNAs, thereby limiting their post-transcriptional expression. Downregulation of let-7 relieves this repression, resulting in elevated cytokine production and amplification of inflammatory responses. Beyond direct cytokine regulation, miRNAs can also modulate inflammatory signaling through metabolic–epigenetic mechanisms. Subsequent work by Jiang et al. further elucidated the molecular basis of let-7 mediated regulation [42]. Using macrophages derived from knockout and transgenic mice, together with in vivo *Salmonella* infection models, the authors demonstrated that the let-7adf cluster directly suppresses the DNA demethylase Tet2, a negative regulator of interleukin-6 (IL-6) production in macrophages [43]. Enhanced let-7adf expression or Tet2 deficiency significantly increased IL-6 induction following LPS or *Salmonella* challenge. In addition, let-7adf promoted intracellular succinate accumulation through regulation of the Lin28a–SDHA axis. Because succinate inhibits Tet2 activity, this metabolic–epigenetic feedback loop further potentiated IL-6 expression. Additional miRNAs contribute to inflammatory control through similar mechanisms. Xu reported that *Salmonella* infection of RAW264.7 macrophages markedly downregulated miR-139-5p, resulting in derepression of its target TRAF6 and activation of both NF-κB and MAPK pathways [44]. This activation promoted robust production of pro-inflammatory cytokines, including IL-1β and TNF-α. Conversely, miR-139-5p overexpression attenuated cytokine secretion and alleviated oxidative stress by increasing the activities of superoxide dismutase, catalase, and glutathione peroxidase while reducing malondialdehyde accumulation [45]. In porcine ileum infected with *Salmonella* Typhimurium, miRNA profiling identified miR-194a-5p as one of the most strongly downregulated species [46]. Because miR-194a-5p directly targets TLR4, its reduction resulted in enhanced receptor expression, activation of downstream inflammatory signaling, and increased pro-inflammatory cytokine production. Similarly, integrated transcriptomic analyses in chicken infection models revealed pronounced upregulation of miR-20b-5p, which directly targets SCNN1A and is closely associated with enrichment of TLR-related signaling pathways [47]. In addition, Sun et al. demonstrated that miR-1306-5p is significantly upregulated during *Salmonella* enteritidis infection and directly targets Toll-interacting protein (Tollip), a negative regulator of TLR signaling. Suppression of Tollip promoted NF-κB activation and increased production of TNF-α, IL-6, and IL-1β, thereby enhancing host resistance to infection [48].

Beyond receptor-level regulation, miRNAs modulate key adaptor proteins that transmit signals from pattern-recognition receptors to downstream pathways such as NF-κB, thereby shaping inflammatory responses. Kang et al. demonstrated that *Salmonella* flagellin stimulation or infection with live bacteria significantly reduced miR-5112 expression in murine bone marrow derived dendritic cells and splenic dendritic cells [49]. Overexpression of miR-5112 suppressed production of IL-6, IL-12p40, and TNF-α, whereas miR-5112 inhibition enhanced inflammatory responses. Mechanistically, miR-5112 directly binds the 3′UTR of IKKγ, thereby attenuating TLR5-mediated NF-κB activation [50]. In vivo administration of a miR-5112 agomir reduced tissue inflammation, lowered bacterial burden, and delayed mortality in infected mice, highlighting its immunomodulatory potential. miR-126-5p has also been shown to regulate adaptor-mediated signaling by directly targeting TRAF3, a central component of both TLR and RIG-I like receptor (RLR) pathways. By suppressing TRAF3 expression, miR-126-5p modulates the IKKε/TBK1–IRF3 axis and influences type I interferon production [51]. Consistent with this mechanism, Mirafzali et al. observed upregulation of gga-miR-126-5p in chickens following *Salmonella* infection, suggesting a conserved role in fine-tuning interferon-dependent innate immunity [52].

miRNAs play an important role in regulating interferon and RIG-I like receptor (RLR) signaling pathways during *Salmonella* infection. Although *Salmonella* is a bacterial pathogen, interferon-related signaling has emerged as an important component of host defense. Using miRNA profiling in infected chicken cecal tissues, Hu et al. reported no significant change in total miR-146b-5p abundance; however, multiple miR-146b-5p isomiRs increased approximately two-fold during infection [53]. These isomiRs directly suppressed the deubiquitinase USP3, thereby relieving inhibition of melanoma differentiation associated protein 5 (MDA5) and sustaining RLR signaling. Activation of this miR-146b-5p isomiR–USP3–MDA5 axis ultimately enhanced type I interferon production, revealing an additional layer of post-transcriptional regulation mediated by miRNA sequence heterogeneity.

Exosomal miRNAs serve as an important mechanism of intercellular immune communication during *Salmonella* infection. Emerging evidence increasingly indicates that infection not only alters intracellular miRNA expression but also reshapes the miRNA cargo of host-derived exosomes. In RAW264.7 macrophages, infection resulted in pronounced enrichment of miR-27a-5p within secreted exosomes. These exosomes could be internalized by neighboring uninfected macrophages, where exosomal miR-27a-5p directly targeted TLR7 and suppressed NF-κB signaling, leading to reduced expression of IL-6 and IL-1β [54]. Further mechanistic analysis demonstrated that selective loading of miR-27a-5p into exosomes depended on the RNA-binding protein hnRNP A/B. *Salmonella* infection upregulated hnRNP A/B expression and promoted preferential packaging of miR-27a-5p, whereas hnRNP A/B deficiency markedly reduced its exosomal abundance [55]. Together, these findings delineate an hnRNP A/B–miR-27a-5p–TLR7/NF-κB regulatory axis that facilitates immune evasion through intercellular communication.

Certain miRNAs contribute to intracellular survival and bacterial persistence during *Salmonella* infection. While most *Salmonella*-responsive miRNAs modulate host inflammatory signaling, a limited subset directly influences bacterial replication and persistence within host cells. In a porcine infection model, Huang et al. identified significant downregulation of miR-143 in macrophages following infection [56]. Subsequent functional analyses revealed that miR-143 directly targets ATP6V1A, a key component of the vacuolar ATPase complex [57]. Reduced miR-143 expression increased ATP6V1A levels, enhanced endosomal activity, and promoted intracellular *Salmonella* replication. Conversely, restoration of miR-143 expression restricted bacterial persistence, highlighting its role in determining infection outcome.

Collectively, studies across multiple experimental systems indicate that miRNA-mediated regulation during *Salmonella* infection is predominantly centered on intrinsic control of innate immune signaling strength rather than on direct bactericidal activity. Conserved miRNAs, including members of the let-7 and miR-146 families, function as immune rheostats that fine-tune inflammatory amplitude and duration, whereas context-dependent miRNAs—particularly those enriched in exosomes—enable *Salmonella* to manipulate intercellular communication and immune thresholds. These multilayered regulatory networks underscore the importance of miRNAs in shaping host–pathogen interactions and provide a conceptual framework for understanding post-transcriptional immune regulation during intracellular bacterial infection. To improve readability and facilitate cross-study comparison, key miRNA–target interactions, associated signaling pathways, experimental models, and functional outcomes reported during *Salmonella* infection are summarized in Table 1.

### 3.2. MicroRNA-Mediated Control of Intracellular Checkpoints and Cytokine Signaling During Listeria monocytogenes Infection

*Listeria monocytogenes* (LM) is a Gram-positive facultative intracellular pathogen that invades host cells through Internalin family proteins (InlA and InlB) and relies on key virulence determinants, including listeriolysin O (LLO) and ActA, to mediate phagosomal escape, intracellular replication, and cell-to-cell dissemination. These virulence strategies enable LM to cause invasive listeriosis, which manifests clinically as severe systemic infections such as sepsis, meningoencephalitis, and pregnancy-associated disease [58,59,60]. During infection, LM activates multiple host immune signaling pathways, including nuclear factor κB (NF-κB), mitogen-activated protein kinase (MAPK), phosphoinositide 3-kinase (PI3K)/Akt, and interferon-related cascades. Increasing evidence indicates that these pathways are tightly regulated by host microRNAs (miRNAs), including miR-155, miR-146a, and miR-21 [61]. In contrast to acute extracellular bacterial infections, immune control of LM requires a finely tuned balance between effective intracellular defense and limitation of immunopathology. Accordingly, miRNAs associated with LM infection primarily function to coordinate inflammatory amplitude, tissue protection, and adaptive immune programming, rather than directly determining bacterial killing efficiency. The following section summarizes current advances in understanding miRNA-mediated immune regulation during LM infection. Mechanistic insights into LM-induced miRNA regulation are mainly supported by murine macrophage infection models and in vivo studies, with emerging evidence highlighting miRNA involvement in adaptive immune programming during invasive listeriosis.

miRNAs play a critical role in regulating early intracellular checkpoints in macrophages during *Listeria monocytogenes* infection. The initial interaction between LM and macrophages represents a key determinant of infection outcome. Following internalization, bacterial survival depends on efficient escape from the phagosome and establishment of cytosolic replication. Several miRNAs have been identified as regulators of these early intracellular checkpoints. Zhang et al. reported that miR-26a, miR-27a, and miR-196b were significantly downregulated in murine bone marrow derived macrophages following LM infection. Functional analyses revealed that miR-26a directly targets ephrin receptor tyrosine kinase A2 (EphA2). Suppression of EphA2 impaired phagosomal escape of LM, thereby limiting cytosolic replication and intracellular survival within macrophages [62]. Consistent with a role in regulating pathogen entry and intracellular permissiveness, previous studies demonstrated that miR-21 restricts macrophage uptake of LM [63]. Luo et al. further showed that miR-21a expression is markedly upregulated following LM infection [64]. Mechanistically, miR-21a directly targets programmed cell death 4 (PDCD4), a gene originally identified as apoptosis-associated and widely recognized as a tumor suppressor. Downregulation of PDCD4 activates the pro-inflammatory c-Jun/STAT3 signaling axis, which contributes to suppression of bacterial infection. Collectively, these findings indicate that specific miRNAs operate at early stages of LM infection to regulate bacterial uptake, phagosomal escape, and cytosolic replication, thereby shaping the permissiveness of macrophages to intracellular colonization.

miRNAs play an important role in regulating interferon- and cytokine-mediated signaling through the SOCS–JAK–STAT pathway. Interferon- and cytokine-mediated signaling is essential for restricting intracellular pathogens but must be tightly controlled to avoid excessive inflammation and maintain immune homeostasis. miRNA-mediated regulation of suppressor of cytokine signaling (SOCS) proteins represents a central mechanism for fine-tuning these pathways during LM infection. Through reanalysis of macrophage transcriptomic datasets, Mishra et al. identified miR-30e-5p as significantly upregulated upon LM infection. This miRNA directly targets SOCS1 and SOCS3, two key inhibitory regulators of the JAK–STAT pathway. By suppressing SOCS1/3 expression, miR-30e-5p enhances interferon and cytokine signaling, limits bacterial proliferation, and contributes to effective antibacterial defense [65]. A parallel regulatory mechanism operates during neuroinvasive LM infection. In the early stages of central nervous system invasion, interferon-γ signaling induces robust upregulation of miR-155 in microglia. miR-155 directly targets SOCS1, thereby relieving inhibition of JAK–STAT signaling and sustaining a pro-inflammatory microglial phenotype [66]. This signaling environment promotes chemokine production, facilitates recruitment of peripheral CD8^+^ T cells into the brain, and supports their differentiation into tissue-resident memory T cells. Together, these studies demonstrate that miRNA-mediated modulation of the SOCS–JAK–STAT axis constitutes a conserved regulatory strategy that amplifies protective immunity while simultaneously predisposing tissues to prolonged inflammatory signaling under certain contexts.

miRNAs constrain inflammatory magnitude by modulating the PTEN–PI3K–Akt–GSK3 signaling pathway. While robust inflammatory responses are necessary for intracellular pathogen control, excessive cytokine production can result in tissue damage. Several miRNAs function to restrain immune activation by regulating metabolic and signaling pathways that govern inflammatory amplitude. Zhang et al. demonstrated that three paralogous miRNA clusters within the miR-17~92 family (miR-17~92, miR-106a~363, and miR-106b~25) act cooperatively to suppress translation of phosphatase and tensin homolog (PTEN). PTEN is a negative regulator of the PI3K–Akt pathway; thus, its suppression leads to enhanced Akt phosphorylation and downstream inhibition of glycogen synthase kinase 3 (GSK3). Because GSK3 promotes interleukin-12 (IL-12) expression, its inhibition results in reduced IL-12 production [67]. These findings indicate that, in wild-type hosts, the miR-17~92 family constrains inflammatory magnitude by limiting IL-12 driven immune amplification, thereby preventing excessive inflammation and maintaining immune homeostasis during LM infection.

Exosomal and ceRNA-mediated mechanisms constitute an important layer of intercellular immune regulation during *Listeria monocytogenes* infection. Beyond intracellular signaling pathways, LM infection also reshapes intercellular immune communication through modulation of exosomal cargo and competing endogenous RNA (ceRNA) interactions. Jiao et al. proposed that LM selectively interferes with host transcription or virulence-associated pathways to reduce the abundance of Rpl13a-213 during exosome biogenesis in macrophages [68]. This reduction weakens the sponge-like sequestration of miR-132-3p, thereby increasing the availability of free miR-132-3p. The released miRNA suppresses inflammatory signaling pathways such as NF-κB, reduces pro-inflammatory cytokine production, and promotes macrophage polarization toward an anti-inflammatory M2 phenotype. This exosome-associated regulatory mechanism creates a more permissive intracellular niche for bacterial persistence and highlights an additional layer of immune modulation that operates beyond cell-autonomous miRNA activity.

During *Listeria monocytogenes* infection, miRNA-mediated regulation extends beyond innate immunity to program adaptive immune responses and shape long-term pathological outcomes. Wheeler et al. demonstrated that the long non-coding RNA Malat1 functions as a molecular sponge for miR-15/16, thereby relieving repression of critical target genes such as CD28 and Bcl-2 [69]. This regulatory axis enhances CD8^+^ T-cell activation, promotes interleukin-2 secretion, and supports the persistence of memory T cells, revealing an additional layer of post-transcriptional control over adaptive immune programming. In neuroinvasive listeriosis, sustained miR-155 expression plays a central role in linking acute host defense to chronic pathology. Persistent miR-155–SOCS1 signaling maintains chemotactic networks that support long-term accumulation of tissue-resident memory T cells in the brain [70]. Experimental antagonism of miR-155 significantly reduces pathological immune cell persistence, underscoring its functional importance [71]. With aging, dysregulated miR-155 expression leads to derepression of the transcription factor C/EBP, aberrant infiltration of pro-inflammatory myeloid cells, and markedly exacerbated neural tissue injury. This non-resolving inflammatory environment ultimately drives synaptic damage and progressive cognitive impairment, with disease severity correlating closely with residual T-cell burden in the brain [72]. Complementing these observations, Liu et al. demonstrated that miR-23a expression in CD4^+^ T cells is dynamically regulated across effector expansion, contraction, and memory formation phases during LM infection [73]. miR-23a directly targets peptidyl-prolyl cis–trans isomerase F (PPIF), a key regulator of the mitochondrial permeability transition pore. By limiting cytosolic reactive oxygen species flux and preserving mitochondrial integrity, miR-23a mitigates excessive inflammation and protects against severe liver injury, thereby supporting systemic immune homeostasis.

Collectively, miRNA-mediated regulation during *Listeria monocytogenes* infection exhibits pronounced cell-type specificity and infection-stage dependency. While core miRNAs such as miR-155 and miR-21 participate in conserved inflammatory feedback circuits, additional miRNAs preferentially modulate PI3K–Akt signaling, JAK–STAT pathways, mitochondrial integrity, and adaptive immune memory formation. These regulatory networks primarily function to balance protective immunity with prevention of immune-mediated tissue damage rather than to directly dictate bacterial clearance. Understanding how miRNAs coordinate innate and adaptive immunity across distinct cellular compartments will be essential for defining host–pathogen equilibrium during intracellular infection and may provide a rational molecular framework for therapeutic intervention in severe or chronic listeriosis. To improve readability and facilitate comparison across studies, key miRNA–target interactions, associated signaling pathways, experimental models, and functional outcomes reported during *Listeria monocytogenes* infection are summarized in Table 2.

### 3.3. MicroRNA-Driven Multilayer Immune Reprogramming During Mycobacterium tuberculosis Infection

*Mycobacterium tuberculosis* (Mtb) is a Gram-positive bacterium that primarily enters the host lung through inhalation of aerosolized droplet nuclei. Following entry, Mtb is phagocytosed by alveolar macrophages or invades type II alveolar epithelial cells, where it establishes a protected intracellular niche. Through specialized secretion systems and multiple virulence-associated factors, Mtb interferes with phagosome maturation and host immune signaling pathways, thereby enabling long-term intracellular persistence [74,75,76,77]. Clinically, these processes manifest as active pulmonary tuberculosis characterized by chronic cough, fever, night sweats, weight loss, and progressive lung tissue destruction, whereas a large proportion of infected individuals remain asymptomatic in a latent state. Accumulating evidence indicates that miRNAs play central roles in shaping host immune responses during mycobacterial infection. Among bacterial pathogens, Mtb exhibits the most extensive and convergent miRNA-mediated immune reprogramming. Rather than eliciting isolated miRNA responses, Mtb consistently reshapes host miRNA networks targeting inflammatory signaling, immunometabolic pathways, antigen presentation, and adaptive immune regulation. These coordinated miRNA programs collectively generate an intracellular environment permissive for persistent infection. Mtb-driven miRNA remodeling has been characterized using macrophage and epithelial infection models, animal studies, and patient-derived samples, enabling linkage of mechanistic regulation with clinical disease phenotypes.

Conceptually, current evidence suggests that Mtb-associated miRNA programs converge on several core immunoregulatory modules. These include suppression of TLR/NF-κB and MAPK-driven inflammatory signaling, metabolic rewiring that weakens bactericidal effector functions, epitranscriptomic control of miRNA maturation (notably via m6A modification), and reprogramming of antigen presentation and adaptive immunity. In the following sections, we summarize these mechanisms in a pathway-oriented manner to highlight how distinct miRNAs collectively shape an intracellular niche permissive for mycobacterial persistence.

miRNAs modulate canonical inflammatory signaling pathways during *Mycobacterium tuberculosis* infection. Cui et al. demonstrated that miR-20a-3p is significantly upregulated in Mtb-infected RAW264.7 macrophages and bone marrow derived macrophages (BMDMs). miR-20a-3p directly targets IKKβ, thereby inhibiting NF-κB activation and reducing secretion of IL-1β, IL-6, and TNF-α, ultimately facilitating intracellular mycobacterial persistence [78]. Similarly, Liu et al. reported marked induction of miR-502-3p following macrophage infection with Mtb. By targeting Rho-associated coiled-coil-containing protein kinase 1 (ROCK1), miR-502-3p suppresses the TLR4/NF-κB signaling axis and attenuates inflammatory mediator production, promoting bacterial survival within macrophages [79].

Additional miRNAs converge on upstream NF-κB regulators. Li et al. observed that miR-140 is strongly induced during Mtb infection and directly targets TRAF6, thereby suppressing TRAF6-dependent NF-κB activation and reducing levels of IL-6, TNF-α, IL-1β, and IFN-γ [80]. In both active and latent tuberculosis patients, Zhang et al. identified significant upregulation of miR-1236-3p, which directly binds the 3′UTR of TLR4, inhibiting TLR4–MyD88–NF-κB signaling and impairing ROS and NO generation in macrophages [81]. Beyond miRNA–mRNA interactions, noncoding RNA crosstalk can further tune NF-κB signaling: Luo et al. showed that Mtb infection induces lncRNA XIST and suppresses miR-125b-5p expression. Acting as a competing endogenous RNA, XIST relieves miR-125b-5p mediated repression of A20, thereby dampening NF-κB activation and promoting an immunoregulatory macrophage phenotype [82]. Notably, mycobacterial virulence factors can also directly manipulate host miRNA expression. Chen et al. reported that the mycobacterial virulence-associated protein Rv0222 suppresses host miR-9 expression, resulting in increased SIRT1 levels and attenuation of NF-κB (p65) signaling, which collectively reduces proinflammatory cytokine production and favors intracellular mycobacterial survival [83].

Mtb-induced miRNA regulation also involves MAPK pathways. Jahan et al. reported that miR-17-5p is markedly upregulated in PMA-differentiated THP-1 macrophages and in macrophage-derived exosomes following infection. miR-17-5p directly suppresses MAP3K2 expression, leading to reduced activation of ERK, JNK, and p38 MAPK pathways and attenuated production of TNF-α, IL-6, IL-1β, inducible nitric oxide synthase, and reactive oxygen species [84]. In contrast, Zhu et al. found that miR-18b-5p is significantly downregulated in infected THP-1 and RAW264.7 macrophages, leading to derepression of HIF-1α. Elevated HIF-1α activates p38 MAPK and NF-κB p65 signaling, enhances pro-inflammatory cytokine expression, and improves macrophage clearance of Mtb [85]. In line with the importance of multilayer RNA regulation, Zhang et al. further showed that circ-WDR27 can sponge miR-370-3p to derepress FSTL1, thereby modulating inflammatory cytokine secretion and mycobacterial vitality in infected macrophages [86].

Collectively, these findings highlight that Mtb-induced miRNA networks preferentially target upstream signaling hubs (e.g., TLR4, TRAF6, IKKβ, and MAP3K2), thereby dampening NF-κB/MAPK activation and limiting pro-inflammatory cytokine output. This regulatory strategy represents a recurrent immune-evasion axis that raises the threshold required for effective mycobacterial clearance.

miRNAs regulate immunometabolic pathways and antimicrobial effector functions during *Mycobacterium tuberculosis* infection. Beyond inflammatory signaling, miRNAs profoundly influence macrophage metabolic reprogramming and antimicrobial effector mechanisms, which represent key determinants of intracellular mycobacterial control. Mal et al. reported that miR-26a expression is reduced in Mtb-infected macrophages, whereas its target, the histone deacetylase associated regulator SIRT6, is upregulated. Increased SIRT6 suppresses inducible nitric oxide synthase (iNOS) as well as IL-6 and IL-1β production, while enhancing expression of the anti-inflammatory enzyme arginase, thereby creating a metabolic environment favorable for mycobacterial survival [87]. Metabolic reprogramming is further mediated by miR-21, which is persistently induced during chronic Mtb infection. Hackett et al. demonstrated that miR-21 directly targets phosphofructokinase muscle type (PFK-M), a rate-limiting glycolytic enzyme. Suppression of glycolytic flux diminishes HIF-1α dependent IL-1β production and reduces NO and ROS generation, resulting in impaired macrophage bactericidal activity and enhanced intracellular replication of Mtb [88]. In contrast, certain miRNAs enhance antimicrobial responses. Fu et al. showed that miR-342-3p is downregulated during Mtb infection, whereas restoration of miR-342-3p expression significantly improves bacterial control. Mechanistically, miR-342-3p targets suppressor of cytokine signaling 6 (SOCS6), thereby relieving inhibition of JAK/STAT1 signaling and increasing expression of TNF-α, IL-1, IL-6, and multiple chemokines [89].

Epitranscriptomic mechanisms play an important role in regulating miRNA biogenesis during *Mycobacterium tuberculosis* infection. Emerging evidence indicates that Mtb modulates host immune responses not only by altering miRNA expression but also by reprogramming miRNA biogenesis through m6A-dependent RNA modifications. Ma et al. reported that Bacillus Calmette–Guérin (BCG) infection reduces expression of the m6A methyltransferase METTL3 in THP-1 derived macrophages, leading to impaired maturation and decreased abundance of miR-29a-3p. Reduced miR-29a-3p relieves suppression of inflammatory signaling and increases secretion of IL-1β, IL-6, and TNF-α [90]. Feng et al. further demonstrated that compromised METTL14-mediated m6A modification similarly reduces miR-29a-3p levels, resulting in enhanced MAP2K6-driven MAPK signaling and downstream NF-κB activation [91]. Consistently, Zhu et al. integrated spinal tuberculosis lesion tissues with a BCG-infected macrophage model and found that the m6A demethylase ALKBH5 is upregulated in the inflammatory microenvironment. ALKBH5 reduces m6A modification on pri-miR-29a-3p, inhibits its maturation, and promotes expression of IL-1β, TNF-α, and IL-17A, thereby amplifying inflammatory responses during infection [92].

During tuberculosis, miRNAs play an important role in regulating adaptive immunity and immune checkpoint pathways, extending their immunomodulatory functions beyond innate immune responses. Zhang et al. demonstrated that interferon regulatory factor 7 (IRF7) signaling is essential for maintaining the PD-1/PD-L1 axis and downstream expression of miR-31 in Mtb-infected mice. Disruption of IRF7 signaling or PD-L1 blockade markedly reduced miR-31 levels, resulting in excessive pulmonary cytokine production, impaired T-cell effector and memory differentiation, and increased mycobacterial burden [93]. Using a BCG-stimulated immature dendritic cell (imDC)–CD4^+^ T-cell co-culture model, Zhen et al. showed that mycobacterial stimulation induces miR-99b expression. miR-99b suppresses mTOR signaling, reduces Th17 differentiation, enhances Treg development, and downregulates IL-6, IL-17, and IL-23 production, thereby generating an adaptive immune milieu favorable for immune evasion by mycobacteria [94]. Furthermore, Chen et al. reported that miR-23a-3p directly targets the transcription factors SP1 and IRF1, preventing excessive activation of both the TLR4/TNF-α pro-inflammatory pathway and the IL-10/TGF-β1 immunosuppressive pathway. Through this dual regulation, miR-23a-3p contributes to restoration of immune homeostasis during Mtb infection [95].

Mtb-induced miRNA regulation is not confined to macrophages but extends to non-professional phagocytes, particularly epithelial cells, and contributes to miRNA-mediated intercellular communication. Using an A549 type II alveolar epithelial cell model, Cui et al. demonstrated that plasma-derived exosomes from patients with active tuberculosis deliver miR-766-3p to epithelial cells, while Mtb infection further induces its expression. miR-766-3p directly targets the antimicrobial transporter NRAMP1, thereby increasing intracellular bacterial load [96]. Similarly, Meng et al. reported that miR-4687-5p is significantly induced in Mtb H37Ra infected A549 cells and suppresses NRAMP1 expression, impairing intracellular metal restriction mechanisms essential for mycobacterial control [97]. Conversely, Zhang et al. found that miR-340-5p is markedly downregulated following infection with virulent Mtb H37Rv. Restoration of miR-340-5p expression reduces intracellular bacterial survival by targeting TMED7 and attenuating TMED7-mediated NF-κB activation, thereby limiting inflammatory signaling associated with persistent infection [98].

From a pathway-level perspective, Mtb-induced miRNA remodeling represents a coordinated multilayer immunosuppressive program that dampens TLR–NF-κB/MAPK-driven inflammatory signaling, rewires glycolysis and nitric oxide related metabolism, and is further reinforced by m6A-dependent regulation of miRNA maturation, collectively weakening macrophage bactericidal activity. Meanwhile, miRNA-mediated modulation of immune checkpoints and T-cell differentiation promotes long-term immune adaptation and persistence, whereas exosome-mediated transfer extends this regulatory network to non-professional phagocytes, including epithelial cells.

Taken together, although individual miRNAs target diverse signaling molecules, their functional consequences during Mtb infection are remarkably convergent. A broad spectrum of miRNAs repeatedly attenuates bactericidal thresholds by suppressing inflammatory signaling, metabolic activation, and antimicrobial effector functions. This convergence supports a model in which diverse miRNAs generate functionally aligned immunosuppressive outcomes that favor persistence. To enhance integrative interpretation of the complex regulatory networks described above, experimentally supported miRNA–target–pathway axes identified in different model systems during *Mycobacterium tuberculosis* infection are summarized in Table 3.

### 3.4. MicroRNA-Mediated Feedback Networks Driving Chronic Inflammation and Immune Evasion During Helicobacter pylori Infection

*Helicobacter pylori* (HP) is a Gram-negative bacterium that predominantly colonizes the human gastric mucosa and represents a major etiological agent of chronic gastritis, gastric ulcer, and duodenal ulcer [99]. Approximately half of the global population is infected with *H. pylori*, with a markedly higher prevalence in developing regions. Persistent infection substantially increases the risk of gastric cancer and mucosa-associated lymphoid tissue (MALT) lymphoma [100]. Unlike acute invasive pathogens, *H. pylori* establishes long-term extracellular colonization at the gastric mucosal surface. This colonization induces sustained but relatively low-grade immune activation rather than rapid bacterial clearance. Consequently, host immune responses are characterized by chronic inflammatory remodeling that requires precise regulatory control to limit tissue injury while permitting bacterial persistence. In this context, microRNAs (miRNAs) have emerged as central post-transcriptional regulators responsible for maintaining gastric immune homeostasis. Evidence for *H. pylori* associated miRNA dysregulation spans gastric epithelial and immune cell infection models, animal studies, and human gastric biopsy datasets, supporting both mechanistic interpretation and biomarker relevance.

TLR–NF-κB signaling serves as a central platform for miRNA induction during *Helicobacter pylori* infection. Recognition of *H. pylori* derived pathogen-associated molecular patterns by pattern recognition receptors, particularly Toll-like receptors (TLRs), initiates innate immune activation. These pathways coordinate the expression of inflammatory mediators while simultaneously inducing immune-regulatory miRNAs. Early work by Xiao et al. demonstrated that *H. pylori* infection markedly upregulates miR-155 in gastric epithelial cells through NF-κB and AP-1 dependent mechanisms, thereby modulating IL-8 production and growth-related gene expression [101]. Consistently, Cortés-Márquez et al. reported that both miR-155 and miR-146a are significantly induced in macrophages from infected individuals. These miRNAs suppress inflammatory signaling through the AP-1/NF-κB pathway and the IRAK1/TRAF6 axis, respectively, forming intrinsic negative feedback circuits that restrict excessive immune activation [102]. Collectively, these findings establish the TLR–NF-κB pathway as the central signaling platform linking *H. pylori* recognition to miRNA-mediated immune regulation.

During chronic *Helicobacter pylori* infection, gastric immune homeostasis is maintained by core miRNA feedback networks dominated by miR-155 and miR-146a. miR-155 is consistently upregulated in infected gastric epithelial cells and immune populations and is closely associated with immune cell activation and chronic inflammation maintenance [103]. In addition to its intracellular functions, *H. pylori* infected macrophages release exosomes enriched in miR-155, facilitating intercellular dissemination of inflammatory signals. Exosomal miR-155 enhances activation of the MyD88–NF-κB pathway and promotes inflammatory mediator release, thereby contributing to antibacterial immune responses [104]. Nevertheless, sustained miR-155 expression may aggravate mucosal injury, underscoring its dual role in host defense and inflammatory pathology. In contrast, miR-146a functions predominantly as a classical immune-negative regulator. By directly targeting IRAK1 and TRAF6, miR-146a attenuates prolonged TLR-mediated NF-κB activation and limits cytokine overproduction. Li et al. further demonstrated that miR-146a suppresses IL-17A driven inflammatory responses, a mechanism particularly relevant to Th17-associated gastric inflammation during *H. pylori* infection [105]. Comprehensive analyses summarized by Zhou et al. revealed that miR-155 and miR-146a jointly regulate NF-κB signaling, Th1/Th17 differentiation, and cAMP activity, thereby maintaining immune stability under conditions of persistent bacterial colonization [106].

Beyond the core miRNA circuitry, expanded regulatory networks composed of multiple auxiliary miRNAs fine-tune inflammatory responses during *Helicobacter pylori* infection. Yang et al. reported that miR-223-3p is significantly upregulated in infected macrophages and suppresses inflammatory signaling by targeting IRAK1 and ARID1A [107]. Similarly, Wang et al. demonstrated that miR-223 overexpression inhibits the production of TNF-α, IL-6, and IL-1β, confirming its anti-inflammatory regulatory function [108]. At a broader level, the let-7 family, miR-125, miR-21, and miR-221 modulate immune responses primarily through the TLR–NF-κB axis. These miRNAs collectively influence cytokine expression and immune cell function and have been proposed as potential biomarkers for *H. pylori* infection and related gastric lesions, including gastric cancer [109,110]. In pediatric gastric mucosa, expression levels of miR-146a and miR-125b correlate closely with infection severity, highlighting the conserved clinical relevance of miRNA-mediated immune regulation across age groups [111]. Consistent with these experimental observations, multiple independent reviews have emphasized that *H. pylori* is capable of actively manipulating host miRNA networks—including miR-155, miR-146a, and the let-7 family—to modulate innate and adaptive immune responses, thereby facilitating long-term colonization and immune evasion [109,110,111,112]. Taken together, these expanded miRNA networks regulate inflammatory magnitude rather than initiating immune activation.

miRNA dysregulation plays a central role in linking chronic inflammation to gastric carcinogenesis during *Helicobacter pylori* infection. During persistent infection, progressive disruption of miRNA regulatory networks promotes chronic inflammation, tissue remodeling, and malignant transformation. Chen et al. demonstrated that *H. pylori* suppresses miR-204 expression in gastric epithelial cells, leading to upregulation of its target gene BIRC2 and sustained activation of NF-κB signaling, which accelerates inflammatory injury and carcinogenic progression [113]. Consistently, reduced miR-204 expression has been associated with MMP9-mediated tissue destruction in pediatric infection-related inflammatory models [114]. Recent studies have also uncovered miRNA-mediated immune evasion mechanisms. Notably, *H. pylori* downregulates miR-4270 in macrophages, resulting in enhanced CD300E expression and reduced surface MHC-II levels, thereby impairing antigen presentation and adaptive immune activation [115]. Moreover, miR-138 has been reported to participate in the regulation of tumor-infiltrating T-cell function and immune checkpoint associated pathways, including PD-1 and CTLA-4 signaling, thereby contributing to immune escape mechanisms in *Helicobacter pylori* associated gastric tumorigenesis [116].

Targeting miRNA-regulated immune pathways offers promising therapeutic opportunities, with accumulating evidence highlighting their substantial translational potential. Inhibition of miR-155 significantly reduces TNF-α and IL-1β secretion and alleviates acute gastric mucosal injury through suppression of NF-κB signaling, underscoring the importance of the miR-155/SOCS1 axis in gastric inflammation control [117]. Network-based analyses further demonstrate that miR-146a is closely associated with inflammation- and immune-related signaling pathways in *H. pylori* associated gastric cancer, reinforcing its potential clinical relevance [118]. Future studies should focus on elucidating mechanisms governing exosomal miRNA trafficking between epithelial and immune cells and on defining interactions between miRNA networks and the gastric microbiota. Integration of single-cell sequencing, spatial transcriptomics, and multi-omics approaches will enable dynamic mapping of miRNA regulatory circuits across different stages of infection. Such analyses are expected to identify key regulatory nodes underlying the functional transition of miRNAs from immune modulation to pathogenic inflammation maintenance, thereby facilitating the development of miRNA-targeted therapeutic strategies. To facilitate mechanistic comparison and improve readability, key miRNA–target interactions, signaling pathways, experimental models, and functional outcomes reported during *H. pylori* infection are summarized in Table 4.

### 3.5. Other Bacterial Pathogens

Current evidence indicates that substantial progress has been made in profiling miRNA expression and elucidating regulatory mechanisms for selected bacterial pathogens. However, across microbial pathogenesis research, miRNA-related studies remain scarce for most bacterial species, or are still limited to the early phase of basic data accumulation. With this in mind, we provide a brief (and necessarily non-exhaustive) overview of the roles of known miRNAs in infections caused by other bacterial pathogens. Many studies have focused on immune-associated miRNAs, particularly the miR-155 family. When macrophages are exposed to inactivated hypervirulent *Klebsiella pneumoniae*-a highly invasive Gram-negative pathogen capable of causing severe sepsis or septic shock and frequently accompanied by acute lung injury-they release exosomes containing diverse molecular components, in which miR-155-5p is markedly enriched. Xu et al.demonstrated in vitro that exosomal miR-155-5p targets mitogen- and stress-activated kinase 1 (MSK1), activates the p38-MAPK pathway, and promotes macrophage M1 polarization and inflammatory responses; conversely, inhibition of miR-155-5p alleviated lung tissue injury [119]. Qin et al. further reported that myeloid miR-155 contributes to host resistance against hepatic K. pneumoniae infection [120]. Chen et al. observed elevated serum miR-155 levels in patients with community-acquired pneumonia caused by Streptococcus pneumoniae, which may be associated with enhanced cellular inflammatory responses [121]. Yang et al. found that infection of astrocytes with *Escherichia coli*-a common Gram-negative bacterium capable of inducing neuroinflammation-upregulated miR-155; notably, miR-146a was also increased in E. coli-infected astrocytes [122]. Mechanistically, miR-155 suppresses TAB2, whereas miR-146a targets IRAK1 and TRAF6; together, they coordinately regulate Toll-like receptor-mediated NF-κB signaling and epidermal growth factor receptor-NF-κB (EGFR-NF-κB) signaling, thereby modulating bacteria-driven neuroinflammation and protecting the central nervous system from further injury. Fu et al. reported that miR-155 is elevated in macrophages following Pseudomonas aeruginosa infection and promotes macrophage apoptosis by inhibiting the PI3K-Akt pathway, a central regulator of cell death, survival, and proliferation [3].

In *Staphylococcus aureus* infection, the contribution of miRNAs has also received increasing attention. Yang et al. reported that miR-146a is upregulated in a mouse model of S. aureus-induced osteomyelitis, reducing osteoblast loss, altering bone remodeling, suppressing inflammatory cytokine production, and inhibiting osteoclastogenesis [123]. Tian et al. observed pronounced overexpression of miR-155 in bronchoalveolar lavage fluid from children with S. aureus pneumonia; miR-155 overexpression promoted differentiation of Th9 cells (a CD4^+^ T-cell subset) by targeting SIRT1 and reduced the numbers of neutrophils and macrophages as well as pro-inflammatory cytokine production after methicillin-resistant S. aureus (MRSA) infection [124]. In subsequent work, they further showed that miR-155 promotes Th17 differentiation by targeting FOXP3, thereby exacerbating inflammation [125]. Jiang et al. found that miR-30a is significantly upregulated in uterine tissues infected with S. aureus; increased miR-30a suppresses the MyD88/Nox2/ROS/NF-κB pathway, thereby attenuating oxidative stress and inflammatory responses triggered by S. aureus lipoteichoic acid (LTA) [126]. Liu et al. reported that miR-127 is upregulated in S. aureus-infected RAW264.7 macrophages as well as in alveolar macrophages (AMs) both in vivo and in vitro [127]. Overexpressed miR-127 suppresses the ubiquitin-editing enzyme A20, thereby promoting K63-linked ubiquitination of STAT3, enhancing macrophage bactericidal activity, and increasing the production of IL-22, IL-17, and antimicrobial peptides.

Despite these advances, important limitations remain. Most studies continue to focus on validating single miRNA-target gene relationships, with limited systems-level analyses. Variability in cell lines and animal models across studies further constrains interpretation and generalizability. In addition, miRNA distribution across tissues and mechanisms of intercellular transport (e.g., exosome-mediated transfer) remain relatively underexplored. Notably, direct mechanistic interactions between miRNAs and bacterial virulence factors have received insufficient attention. Future work should adopt a systems perspective to construct integrated miRNA-target-signaling pathway networks and combine transcriptomic, proteomic, and epigenomic datasets to define miRNA regulatory functions throughout infection. Integration with clinical data will facilitate the development of novel miRNA biomarkers and targeted intervention strategies, providing a stronger theoretical and technical foundation for precision prevention and control of bacterial infections.

Although studies on other bacterial pathogens remain fragmented, a recurring theme is the preferential involvement of conserved immune-associated miRNAs-particularly miR-155 and miR-146a—in tuning inflammatory signaling thresholds across diverse infection contexts. This suggests that microRNA-mediated immune calibration represents a broadly conserved strategy rather than a pathogen-specific phenomenon.

### 3.6. Conceptual Framework: miRNAs as Immune Rheostats in Bacterial Infection

**Figure 2:**
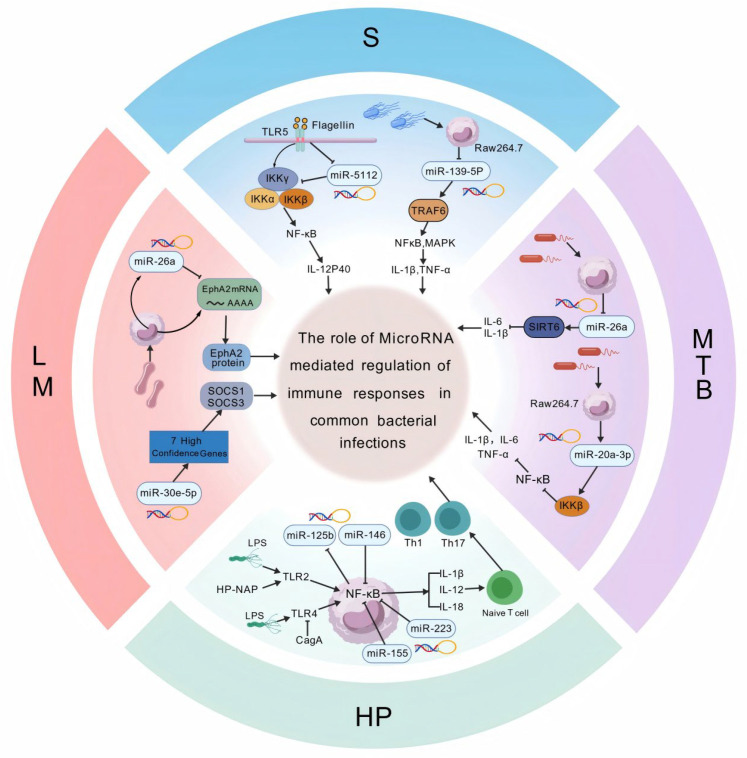
MicroRNA-mediated regulation of immune responses in common bacterial infections. This schematic illustrates representative microRNA-regulated signaling pathways involved in host immune responses to *Salmonella* (S), *Listeria monocytogenes* (LM), *Mycobacterium tuberculosis* (MTB), and *Helicobacter pylori* (HP). Specific miRNAs modulate key innate and adaptive immune pathways, including TLR/NF-κB signaling, cytokine production (IL-1β, IL-6, TNF-α), metabolic reprogramming, and T-cell differentiation. Through targeting transcription factors, signaling adaptors, and metabolic regulators, miRNAs fine-tune inflammatory intensity, immune cell activation, and host pathogen interactions during bacterial infection.

Accumulating evidence across diverse bacterial infection models indicates that miRNAs do not operate as isolated pro- or anti-inflammatory regulators. Instead, they constitute a hierarchically organized immune threshold–control system that calibrates the intensity, duration, and spatial distribution of host immune responses. To integrate these findings across diverse bacterial pathogens, we propose a unified conceptual model in which miRNAs function as hierarchical immune rheostats rather than binary pro- or anti-inflammatory regulators. In this framework, conserved miRNAs establish global immune activation thresholds by targeting central signaling hubs, while context-dependent miRNAs fine-tune pathway utilization, metabolic allocation, and intercellular communication in a spatially and temporally restricted manner. Figure 2 schematically summarizes representative microRNA-mediated regulatory circuits during infections with *Salmonella*, *Listeria monocytogenes* (LM), *Mycobacterium tuberculosis* (Mtb), and *Helicobacter pylori* (HP). By illustrating how specific miRNAs converge on key immune pathways, including TLR/NF-κB signaling, cytokine production, immunometabolic reprogramming, and T-cell differentiation, this figure highlights both the shared regulatory logic and pathogen-specific adaptations underlying microRNA-driven immune calibration. The figure was created with BioGDP.com. 

Within this framework, miRNA-mediated regulation can be functionally stratified into distinct regulatory layers. A limited set of conserved miRNAs—including miR-155, miR-146a, and miR-21—forms a first-layer threshold setting module. These miRNAs recurrently target central immune hubs such as TLR–NF-κB, JAK–STAT, and core immunometabolic pathways, thereby defining global activation boundaries that are largely conserved across pathogens, host cell types, and tissues. Their frequent integration into negative feedback circuits highlights a fundamental role in preventing uncontrolled inflammation while maintaining sufficient antimicrobial competence. Importantly, these miRNAs do not dictate pathogen clearance directly; rather, they determine whether immune activation is permitted to escalate beyond a critical threshold.

Beyond this conserved core, a second layer of context-dependent miRNAs redistributes immune pressure in space and time. These miRNAs exhibit pronounced specificity for cell type, infection stage, tissue environment, or extracellular vesicle mediated transfer. Rather than shifting immune thresholds globally, they fine-tune pathway preference, metabolic allocation, and intercellular communication, thereby shaping localized immune outcomes without disrupting systemic homeostasis. Failure to distinguish this hierarchical organization likely underlies the apparent inconsistencies reported for individual miRNAs across experimental models.

This layered threshold framework provides a unifying explanation for the seemingly paradoxical roles of miRNAs such as miR-155 and miR-21. Divergent functional outcomes do not reflect experimental noise or contradictory biology, but instead arise from differences in baseline immune state, cellular context, and temporal phase of infection. During early infection, miRNA-mediated dampening of inflammation may facilitate immune evasion and intracellular persistence, whereas sustained or dysregulated activity at later stages can drive chronic inflammation, immune exhaustion, or tissue pathology. Thus, miRNA function is best interpreted relative to immune activation thresholds rather than as a fixed pro- or anti-inflammatory property.

Importantly, this conceptualization yields testable predictions. Perturbation of first-layer threshold-setting miRNAs is expected to shift immune activation limits across multiple bacterial infections, largely independent of pathogen identity. In contrast, modulation of context-dependent miRNAs should produce divergent outcomes depending on cell type, tissue, and infection stage. These principles further suggest that combinatorial miRNA modulation may achieve more precise immune control than single-miRNA targeting, providing a rational framework for host-directed therapeutic strategies.

Collectively, this model reframes miRNAs as hierarchical immune rheostats that integrate pathogen sensing, inflammatory signaling, metabolic reprogramming, and immune resolution. By focusing on threshold control rather than binary inflammatory classification, this framework reconciles disparate observations across bacterial infections and offers a conceptual foundation for translating miRNA biology into predictive biomarkers and precision immunomodulatory interventions. To systematically organize the experimental evidence supporting this framework, Table 5 summarizes representative miRNAs involved in immune regulation during common bacterial infections, including their validated targets, associated signaling pathways, and functional outcomes. Rather than providing an exhaustive list, the table emphasizes experimentally supported miRNA–target interactions that recurrently converge on central immune hubs, such as TLR–NF-κB, JAK–STAT, autophagy, and immunometabolic pathways across pathogens.

## 4. Conclusions and Perspectives

Across *Salmonella*, LM, Mtb, and HP infections, miR-155, miR-146a, and miR-21 consistently emerge as conserved regulators of TLR–NF-κB centered immune circuits, supporting their classification as core immune rheostats rather than pathogen-specific effectors. Rather than acting in isolation, these miRNAs integrate inflammatory signaling, metabolic reprogramming, and intercellular communication to calibrate immune thresholds that ultimately determine infection outcomes, including pathogen clearance, persistence, or immunopathology.

A key implication of this synthesis is that miRNA-mediated immune regulation cannot be adequately described using a binary pro- versus anti-inflammatory paradigm. Instead, miRNAs operate within a dynamic, hierarchical regulatory architecture in which first-layer miRNAs establish global activation thresholds, while context-dependent miRNAs shape spatial and temporal response patterns. This perspective provides a unifying explanation for the divergent roles attributed to the same miRNA across different bacterial infections, cell types, and experimental systems.

Importantly, this framework generates several testable predictions. First, perturbation of core rheostat miRNAs such as miR-155 or miR-146a is expected to shift immune activation thresholds across multiple bacterial infections, largely independent of pathogen identity. Second, miRNAs with context-dependent functions should display divergent or even opposing effects when transferred across cell types or infection stages. Third, combinatorial modulation of miRNAs may achieve more precise immune control than single-miRNA targeting, offering a rational basis for host-directed therapeutic strategies.

Looking forward, advancing the field will require a transition from single-miRNA validation toward systems-level and temporally resolved analyses. Integration of single-cell sequencing, spatial transcriptomics, and multi-omics approaches will enable dynamic mapping of miRNA–target–pathway networks across infection stages and tissue microenvironments. In parallel, exosomal and circulating miRNAs represent particularly attractive biomarkers—detectable, traceable, and potentially actionable—for disease staging, prognosis, and treatment-response monitoring.

As miRNA biology continues to intersect with infection immunology, metabolism, and host–microbiota interactions, miRNA-based diagnostics and immunomodulatory interventions are increasingly poised to complement or extend traditional antimicrobial strategies. By framing miRNAs as hierarchical immune rheostats, this review provides a conceptual foundation for both mechanistic discovery and translational innovation in the prevention and control of bacterial infections.

## Figures and Tables

**Table 1:** Key miRNA–target–pathway networks involved in host immune regulation during *Salmonella* infection.

Key miRNA	Validated Target Gene	Pathway/Axis	Cell Type/Model System	Functional Outcome
let-7 family	IL6, IL10	TLR4–NF-κB/cytokine regulation	Murine macrophages; human epithelial cells; murine infection models	Downregulation of let-7 relieves repression of IL6/IL10, increasing cytokine output
let-7adf cluster	Tet2	TLR4 signaling; metabolic–epigenetic regulation of IL-6	Knockout/transgenic mouse macrophages; in vivo mouse infection models	Enhances IL-6 induction through Tet2 suppression and succinate-mediated Tet2 inhibition
miR-139-5p	TRAF6	NF-κB and MAPK activation	RAW264.7 macrophages (in vitro)	Downregulation derepresses TRAF6 → activates NF-κB/MAPK → increases IL-1β, TNF-α
miR-194a-5p	TLR4	TLR4–NF-κB axis	Porcine ileum infection model (in vivo)	Downregulation increases TLR4 expression and inflammatory cytokines
miR-20b-5p	SCNN1A	TLR-related signaling enrichment	Chicken infection model (in vivo transcriptomics)	Upregulation associated with enhanced TLR pathway activation
miR-1306-5p	Tollip	TLR–NF-κB pathway	Infection model (reported during *S. enteritidis* infection)	Targets Tollip (negative regulator) → increases NF-κB activation and cytokines (TNF-α, IL-6, IL-1β)
miR-5112	IKKγ	TLR5–NF-κB signaling	Murine BMD dendritic cells; splenic DCs; mouse infection model	Overexpression suppresses cytokines; agomir reduces inflammation and bacterial burden
miR-126-5p	TRAF3	TBK1–IRF3; type I IFN signaling	Chicken infection model (in vivo)	Suppresses TRAF3 → modulates IRF3 axis and interferon response
miR-146b-5p isomiRs	USP3	RLR signaling (MDA5activation); type I IFN	Chicken cecal tissues (in vivo)	IsomiRs suppress USP3 → sustain MDA5 signaling → increase type I IFN
exosomal miR-27a-5p	TLR7	TLR7–NF-κB signaling	RAW264.7 macrophages + exosome transfer (in vitro)	Exosomal miR-27a-5p suppresses NF-κB and reduces IL-6/IL-1β in recipient macrophages
miR-143	ATP6V1A	SCV acidification/endosomal activity	Porcine macrophages infection model (in vivo)	Downregulation increases ATP6V1A → enhances intracellular replication; restoration restricts persistence

**Table 2:** Key miRNA–target–pathway networks involved in host immune regulation during *Listeria monocytogenes* infection.

Key miRNA	Validated Target Gene	Pathway/Axis	Cell Type/Model System	Functional Outcome
miR-26a	EphA2	Early intracellular checkpoints (phagosomal escape/cytosolic replication)	Murine BMDMs (in vitro infection)	Targeting EphA2 impairs phagosomal escape → limits cytosolic replication and intracellular survival
miR-21/miR-21a	PDCD4	c-Jun/STAT3 signaling (pro-inflammatory antibacterial axis)	Macrophage infection model (LM-infected; murine context implied)	PDCD4 suppression activates c-Jun/STAT3 → suppresses bacterial infection; also reported to restrict uptake
miR-30e-5p	SOCS1, SOCS3	SOCS–JAK–STAT (IFN/cytokine signaling)	Macrophage transcriptomic reanalysis + infection models	Enhances IFN/cytokine signaling → limits bacterial proliferation
miR-155	SOCS1	IFN-γ–JAK–STAT in CNS; chemokine networks	Microglia; neuroinvasive listeriosis context	Sustains pro-inflammatory microglial phenotype; promotes chemokines, CD8^+^ T-cell recruitment and TRM differentiation
miR-17~92 family	PTEN	PTEN–PI3K–Akt–GSK3 → IL-12 control	In vivo/infection-immunology context (wild-type host)	PTEN → Akt → GSK3 inhibition → IL-12; constrains inflammatory magnitude
miR-15/16	CD28, Bcl-2	Adaptive immunity programming (CD8^+^ activation/memory)	T-cell context (CD8^+^); infection model	Enhances CD8^+^ activation, IL-2 secretion, memory T-cell persistence
miR-155	SOCS1	Chronic neuroinflammation/immunopathology	Brain TRM accumulation; aging context	Maintains immune cell persistence; antagonism reduces persistence; aging exacerbates injury
miR-23a	PPIF	Mitochondrial integrity; ROS control; liver injury protection	CD4^+^ T cells across effector→memory phases	Preserves mitochondrial integrity, reduces ROS-driven inflammation, mitigates liver injury

**Table 3:** Pathogen-specific miRNA regulatory networks involved in host immune responses during *Mycobacterium tuberculosis* infection.

Key miRNA	Validated Target Gene	Pathway/Axis	Cell Type/Model System	Functional Outcome
miR-20a-3p	IKKβ	TLR–NF-κB signaling	RAW264.7 macrophages; BMDMs (in vitro)	NF-κB inhibition → IL-1β/IL-6/TNF-α secretion reduced
miR-502-3p	ROCK1	TLR4/NF-κB signaling	Macrophage infection model	Suppresses inflammatory mediator production
miR-140	TRAF6	TRAF6-dependent NF-κB activation	Macrophage infection model	Decreases IL-6/TNF-α/IL-1β/IFN-γ
miR-1236-3p	TLR4	TLR4–MyD88–NF-κB axis; ROS/NO generation	Active & latent TB patients; macrophage context	NF-κB inhibition → ROS/NO impaired
miR-125b-5p	A20	NF-κB suppression via ceRNA	Macrophages; lncRNA XIST axis	Dampens NF-κB activation; immunoregulatory phenotype
miR-9	SIRT1	NF-κB p65 signaling	Macrophage infection; virulence factor Rv0222	Reduces pro-inflammatory cytokines
miR-17-5p	MAP3K2	MAPK pathways (ERK/JNK/p38)	THP-1 macrophages; exosomes	Suppresses MAPK activation → TNF-α/IL-6/IL-1β/ROS/NO reduced
miR-18b-5p	HIF-1α	p38 MAPK & NF-κB activation	THP-1; RAW264.7 macrophages	Enhances inflammatory cytokines and bacterial clearance
miR-370-3p	FSTL1	Cytokine secretion regulation	Macrophage infection model	Modulates cytokine output and mycobacterial vitality
miR-26a	SIRT6	iNOS regulation; IL-6/IL-1β; arginase	Macrophage infection model	Decreases iNOS/cytokines; increases arginase
miR-21	PFK-M	Glycolysis; HIF-1α–IL-1β; NO/ROS	Chronic infection model	Suppresses glycolysis → IL-1β, NO/ROS reduced
miR-342-3p	SOCS6	JAK/STAT1 signaling	Macrophage infection model	Restoring miR-342-3p enhances TNF-α/IL-1/IL-6/chemokines
miR-29a-3p	METTL3/METTL14/ALKBH5	m6A-dependent miRNA biogenesis	THP-1 macrophages; BCG model; spinal TB lesions	Altered miR-29a-3p maturation reshapes inflammatory signaling
miR-31	PD-1/PD-L1 axis via IRF7	Immune checkpoint signaling	Mtb-infected mice	Maintains immune balance; reduction causes cytokine excess and higher burden
miR-99b	mTOR	Th17/Treg differentiation	BCG-stimulated imDC–CD4^+^ co-culture	Suppresses Th17; enhances Treg; reduces IL-6/IL-17/IL-23
miR-23a-3p	SP1, IRF1	TLR4/TNF-α and IL-10/TGF-β balance	Infection model	Prevents excessive inflammatory & immunosuppressive activation
miR-766-3p	NRAMP1	Antimicrobial transporter; metal restriction	A549 epithelial cells; patient plasma exosomes	Increases intracellular bacterial load
miR-4687-5p	NRAMP1	Metal restriction mechanisms	A549 epithelial cells (H37Ra infection)	Suppresses NRAMP1 → impairs intracellular control
miR-340-5p	TMED7	NF-κB activation	A549 epithelial cells; H37Rv infection	Restoration reduces survival via NF-κB attenuation

**Table 4:** Pathogen-specific miRNA regulatory networks involved in host immune responses during *Helicobacter pylori* infection.

Key miRNA	Validated Target Gene	Pathway/Axis	Cell Type/Model System	Functional Outcome
miR-146a	IRAK1, TRAF6	TLR–NF-κB negative feedback	Macrophages from infected individuals; gastric mucosa	Suppresses prolonged NF-κB signaling and cytokine overproduction
miR-223-3p	IRAK1, ARID1A	TLR–NF-κB axis; inflammatory signaling	Infected macrophages	Inhibits inflammatory signaling; reduces cytokine production
miR-204	BIRC2	NF-κB activation; carcinogenesis link	Gastric epithelial cells (infection model)	miR-204 suppression derepresses BIRC2 → sustained NF-κB activation → inflammation and carcinogenic progression
miR-4270	CD300E	Antigen presentation; MHC-II suppression	Macrophages (infection model)	miR-4270 downregulation increases CD300E → reduces surface MHC-II → impairs antigen presentation
miR-155	SOCS1	NF-κB signaling; gastric inflammation	Gastric inflammation model	miR-155 inhibition reduces TNF-α and IL-1β; alleviates mucosal injury

**Table 5:** MiRNAs involved in immune regulation during common bacterial infections.

miRNA	Target/Pathway	Pathogen	Immune Regulatory Function	Ref.
let-7 family (let-7adf)	Tet2 Lin28a IL-6 axis	*Salmonella*	Enhances IL-6 production while maintaining inflammatory balance through dual regulation of Tet2 expression and succinate-mediated metabolic signaling	[42]
miR-139-5p	TRAF6 NF-κB MAPK	*Salmonella*	Suppresses excessive inflammation and oxidative stress by inhibiting TRAF6-mediated NF-κB and MAPK activation	[44]
miR-194a-5p	TLR4	*Salmonella*	Boosts inflammatory signaling: increased TLR4 expression heightens LPS sensitivity and inflammation.	[46]
miR-20b-5p	SCNN1A/Toll-like	*Salmonella*	Indirectly affects Toll-like receptor-related signaling pathways.	[47]
miR-1306-5p	Tollip/NF-κB	*Salmonella*	Enhances host defense: relieves Tollip-mediated negative regulation, promoting NF-κB activation and antimicrobial mediator release.	[48]
miR-5112	IKKγ NF-κB	*Salmonella*	Promotes IL-6 and IL-12p40 production by targeting IKKγ and acts as a negative-feedback regulator downstream of TLR5-flagellin signaling	[50]
miR-126-5p	TRAF3 IRF3-IFN	*Salmonella*	Limits excessive inflammatory responses by modulating TRAF3-dependent interferon signaling and is proposed as a biomarker for food-borne salmonellosis	[51]
miR-146b-5p (isomiRs)	USP3 RLR-MDA5	*Salmonella*	Sustains type I interferon responses through isomiR-specific targeting of USP3, highlighting functional diversity of miRNA isoforms	[53]
miR-27a-5p	TLR7/NF-κB	*Salmonella*	Facilitates immune evasion: exosomal miRNA dampens inflammation in neighboring cells, weakening overall host defense.	[55]
miR-143	ATP6V1A v-ATPase	*Salmonella*	Restricts intracellular bacterial persistence through ATP6V1A-mediated regulation of *Salmonella*-containing vacuole acidification	[57]
miR-26a	EphA2	*Listeria monocytogenes*	Restricts bacterial escape into the cytosol by down-regulating EphA2 and enhancing macrophage antimicrobial activity	[62]
miR-21/miR-21a	PDCD4 c-Jun-STAT3	*Listeria monocytogenes*	Modulates macrophage uptake and inflammatory responses in a context-dependent manner	[64]
miR-30e-5p	SOCS1 SOCS3	*Listeria monocytogenes*	Enhances innate immune responses and limits bacterial replication through suppression of SOCS-mediated negative feedback	[65]
miR-17~92 family	PTEN/PI3K–Akt	*Listeria monocytogenes*	Prevents excessive inflammation: limits IL-12 production to avoid severe tissue damage.	[67]
miR-132-3p	NF-κB	*Listeria* *monocytogenes*	Promotes immunosuppression (M2): inhibits inflammatory signaling and drives macrophage polarization toward M2, benefiting bacterial survival.	[68]
miR-15/miR-16	CD28; Bcl-2	*Listeria* *monocytogenes*	Enhances T-cell memory: supports CD8^+^ T-cell activation and memory formation, improving adaptive immunity.	[69]
miR-23a	PPIF mitochondrial ROS	*Listeria monocytogenes*	Maintains immune homeostasis by controlling mitochondrial ROS flux during T-cell activation	[73]
miR-20a-3p	IKKβ NF-κB	*Mycobacterium tuberculosis*	Inhibits cytokine production and facilitates immune evasion by targeting IKKβ	[78]
miR-502-3p	ROCK1 TLR4-NF-κB	*Mycobacterium tuberculosis*	Dampens inflammatory signaling and enhances bacterial persistence	[79]
miR-140	TRAF6	*Mycobacterium tuberculosis*	Suppresses host defense: blocks NF-κB signaling and lowers pro-inflammatory gene expression.	[80]
miR-1236-3p	TLR4	*Mycobacterium tuberculosis*	Reduces bactericidal efficiency: directly suppresses TLR4, lowering ROS/NO and promoting intracellular survival.	[81]
miR-125b-5p	A20	*Mycobacterium tuberculosis*	Promotes M2 polarization and survival: suppresses NF-κB and pushes macrophages toward an anti-inflammatory phenotype.	[82]
miR-9	SIRT1	*Mycobacterium tuberculosis*	Weakens innate immunity: via SIRT1-mediated suppression of NF-κB, reduces inflammatory cytokines and promotes survival.	[83]
miR-17-5p	MAP3K2/MAPK	*Mycobacterium tuberculosis*	Weakens bactericidal capacity: decreases ROS and NO, impairing macrophage clearance of bacteria.	[84]
miR-18b-5p	HIF-1α	*Mycobacterium tuberculosis*	Enhances inflammatory responses and favors bacterial clearance	[85]
miR-370-3p	FSTL1	*Mycobacterium tuberculosis*	Promotes inflammatory mediator release and suppresses bacterial survival.	[86]
miR-26a	SIRT6 HIF-1α	*Mycobacterium tuberculosis*	Suppresses inflammatory responses and promotes intracellular bacterial survival through metabolic reprogramming	[87]
miR-21	PFK-m/glycolysis	*Mycobacterium tuberculosis*	Metabolic reprogramming supporting survival: suppresses glycolysis (Warburg-like shift), weakening immunometabolic support for killing.	[88]
miR-342-3p	SOCS6	*Mycobacterium tuberculosis*	Compromised host defense: downregulated upon infection; restoring it can enhance inflammation to control bacteria.	[89]
miR-29a-3p	MAP2K6/MAPK	*Mycobacterium tuberculosis*	Exacerbates inflammatory damage: dysregulation may cause excessive inflammation and pathological tissue changes.	[91]
miR-31	IRF7–PD-1/PD-L1–miRNA-31 axis	*Mycobacterium tuberculosis*	Indirectly restricts mycobacterial expansion in vivo.	[93]
miR-99b	mTOR	*Mycobacterium tuberculosis*	Induces immune tolerance: inhibits Th17 differentiation and promotes Treg development, facilitating immune evasion.	[94]
miR-23A-3p	SP1; IRF1	*Mycobacterium tuberculosis*	Restores immune balance: prevents over-skewing toward excessive pro- or anti-inflammatory states.	[95]
miR-766-3p	NRAMP1	*Mycobacterium tuberculosis*	Promotes intracellular growth: disrupts nutritional immunity by weakening metal restriction.	[96]
miR-4687-5p	NRAMP1	*Mycobacterium tuberculosis*	Disrupts nutritional immunity: suppresses NRAMP1, relieving restriction on bacterial metal ions.	[97]
miR-340-5p	TMED7	*Mycobacterium tuberculosis*	Favors a permissive inflammatory milieu: downregulation sustains an infection-supportive environment (overexpression may improve it).	[98]
miR-146a	IRAK1 TRAF6	*Helicobacter pylori*	Limits excessive inflammatory responses and maintains immune homeostasis	[102]
miR-223-3P	IRAK1 ARID1A	*Helicobacter pylori*	Suppresses macrophage activation and pro-inflammatory cytokine production	[107]
miR-204	BIRC2 NF-κB	*Helicobacter pylori*	Promotes inflammation and infection-associated carcinogenesis and is down-regulated during infection	[113]
miR-4270	CD300E	*Helicobacter pylori*	Impaired antigen presentation: reduces MHC-II expression, weakening T-cell recognition and facilitating immune evasion.	[115]
miR-138	PD-1; CTLA-4	*Helicobacter pylori*	Induces T-cell exhaustion: checkpoint molecule signaling suppresses antitumor/anti-infection immunity.	[116]
miR-155	SOCS1 NF-κB	*Helicobacter pylori*	Acts as a central regulator of chronic gastric inflammation by modulating SOCS1-mediated NF-κB signaling	[117]
miR-155-5p	MSK1 p38-MAPK	*Klebsiella pneumoniae*	Promotes macrophage M1 polarization through exosome-mediated regulation	[119]
miR-146a	IRAK1 TRAF6	*Escherichia coli*	Attenuates neuroinflammation in synergy with miR-155	[122]
miR-30a	MyD88 ROS-NF-κB	*Staphylococcus aureus*	Suppresses oxidative stress and inflammation and confers protection in osteomyelitis	[126]

## Data Availability

No new data were created or analyzed in this study. Data sharing is not applicable to this article.
